# Resource Allocation and Outpatient Appointment Scheduling Using Simulation Optimization

**DOI:** 10.1155/2017/9034737

**Published:** 2017-09-25

**Authors:** Carrie Ka Yuk Lin, Teresa Wai Ching Ling, Wing Kwan Yeung

**Affiliations:** ^1^Department of Management Sciences, College of Business, City University of Hong Kong, Kowloon Tong, Hong Kong; ^2^Albers School of Business and Economics, Seattle University, 901-12 Avenue, Seattle, WA 98122, USA

## Abstract

This paper studies the real-life problems of outpatient clinics having the multiple objectives of minimizing resource overtime, patient waiting time, and waiting area congestion. In the clinic, there are several patient classes, each of which follows different treatment procedure flow paths through a multiphase and multiserver queuing system with scarce staff and limited space. We incorporate the stochastic factors for the probabilities of the patients being diverted into different flow paths, patient punctuality, arrival times, procedure duration, and the number of accompanied visitors. We present a novel two-stage simulation-based heuristic algorithm to assess various tactical and operational decisions for optimizing the multiple objectives. In stage I, we search for a resource allocation plan, and in stage II, we determine a block appointment schedule by patient class and a service discipline for the daily operational level. We also explore the effects of the separate strategies and their integration to identify the best possible combination. The computational experiments are designed on the basis of data from a study of an ophthalmology clinic in a public hospital. Results show that our approach significantly mitigates the undesirable outcomes by integrating the strategies and increasing the resource flexibility at the bottleneck procedures without adding resources.

## 1. Introduction

This article explores how supply and demand planning at the tactical and operational level in terms of resource allocation and patient scheduling can be considered simultaneously in an appointment session. A joint study at an ophthalmology outpatient clinic in a public hospital described in [1, 2] has motivated investigation of this integrated planning problem of improving system performance from the patient and staff perspectives. The indirect benefit is to create an opportunity for increasing service capacity and reduce patient access time to the outpatient service. The problem characteristics in the ophthalmology clinic can be generalized for outpatient clinics operating like a multiphase and multiserver queuing system. A large amount of appointment scheduling research tends to focus on one or two services (e.g., a doctor's consultation as the main focus, with a preconsultation procedure as a secondary focus) to derive analytical properties [3]. Simple structure also allows an optimal patient sequence and schedule to be obtained for queuing systems approximated by distribution-free parameters [4, 5]. Multiphase appointment systems involving a large number of patients in a session are found in other practical situations such as outpatient clinics in public medical centers or hospitals [1, 6]. Patients are often categorized by classes with different routings, requiring multiple resources through the outpatient center with many stochastic factors. Stakeholders express their preferences in multiple often conflicting performance measures [7]. The complexity of the system thus increases the difficulty of making good decisions.

Based on the classification of healthcare planning decisions [8], three types of decisions are studied in this article: the resource allocation, block appointment scheduling at the tactical level, and service discipline (selection of the next patient for treatment) at the operational level. The integrated planning of multiple decisions can offer the advantage of modelling their interactive effects in order to find the best combination of decisions. This is a research direction that was pointed out in a recent survey of optimization studies in outpatient appointment systems [9]. Related studies include a single-phase single-server problem in an outpatient procedure center where decisions on the number of patient bookings, sequencing, and daily scheduling were considered together [3]. With uncertainties in procedure duration and attendance rate, the problem is formulated using a stochastic programming model. The analytical properties derived from the optimal solutions are used to develop near-optimal heuristics for solving larger problems. Another study conducted at a radiology department [10] considered both the resource allocation and the appointment scheduling decisions for two CT scanners (resource). Medium-term capacity is adjusted in terms of opening hours, and short-term allocation of timeslots is made between patient groups. As it is difficult to find optimal solutions for this medium- and short-term planning problem, patient scheduling rules resembling the practice at the department (first-come randomly served, first-come-first-served) are combined with static or dynamic allocation plans to be used as benchmarks for comparison with the proposed methods. The current work is similar in that different service discipline will be combined with the tactical level decisions for performance comparison.

Applying both demand and supply strategies is more effective than a one-way strategy. Demand and supply planning strategies considered in healthcare research often involved employing simulation and optimization techniques in different ways. Surveys on the application of simulation to healthcare can be found in [11–13]. In a capacity planning problem in matching new patients to oncologists [14], the demand strategies applied include patient diversion and the development of scheduling rules to assign new patients to oncologists to satisfy the access time tolerance. The supply strategies include determining various mixes of oncologists with different specializations and adding a number of add-on appointments to the normal weekly capacity to meet demands and fulfil new patients' access time tolerance. The specialization of each oncologist in treating one or more types of tumour in [14] is analogous to the qualified set of skills (procedures) of each resource unit in the current study. The supply of resource units are already given, but they are allocated to tasks in their qualified skill set to better match with the demand strategies (appointment scheduling and service discipline). Our work will incorporate the simulation approach into the heuristic optimization framework in order to search for and to evaluate different resource allocations and patient scheduling decisions that need improvement. As multiple bottlenecks may exist in the system and their interactive effects on the system's performance are not easily understood, a computational approach is adopted to continuously find a set of integrated decisions that can improve the multiple objectives. In another study on admission and capacity planning for skin cancer treatment [15], a new one-stop-shop (OSS) concept was tested for its feasibility and the best way to operate the service. Similar to the patient classification in the current study, the capacity analysis in [15] involves identifying treatment profiles (or types) and resources used for new patients. The throughput (or system) time for the treatment of new patients is the target performance measure for reduction in [15] while multiple objectives are considered in our study.

The healthcare environment often faces conflicting objectives in managing the expectations of different stakeholders. Waiting time on the appointment day is of typical concern for scheduled patients. It is defined as the throughput time excluding the sum of procedure times which better represents the non-value-added time in the system. Excessive overtime lowers staff morale and job satisfaction while congestion in the waiting area affects satisfaction of all parties. This work contributes to minimizing these three objectives through combining them into a weighted objective function where the weights are chosen by the user. In the healthcare literature and survey on planning decisions [8], the first two objectives are adopted more commonly. Congestion has been given less attention with some exception such as a local medical center where multiple specialties share the same waiting area [6]. Among the improvement scenarios, the strategy to reschedule patients from more congested specialty sessions to less congested ones was found to reduce the peak congestion most. This strategy has influenced the present design for rescheduling patients from appointment time blocks experiencing a large *undesirabl*e impact (or contributing a large *value* to the objectives) to time blocks with a smaller impact. In other clinics, service locations of the same specialty clinic are dispersed that not all patients wait in the same area for all procedures. The relationship between patient waiting time, server overtime, and congestion in the clinic waiting area is expressed here by a weighted objective function and their trade-off explored using simulation. Past outpatient studies adopting a similar weighted score approach included [7], a study in a large-scale oncology center. To reduce the patient system time and resource overtime in the multifacility system, a weighted score is constructed considering multiple dimensions by day of the week, patient type, facility, and time period. Improvement strategies include changing either the supply or demand expressed as 16 separate factors. While most of the supply factors in [7] consider adding resource of one doctor or one nurse, our study searches for an improved resource allocation plan computationally without adding resources. The patient scheduling decisions are explored more extensively here by automating the search for the (block) appointment schedule. Similar to [7], the change in service discipline is tested. In [7], priority is given to patients requiring appointments in more facilities than those with fewer. We consider an equivalent priority rule, the largest number of successors (LNS), in addition to other priority rules and a new adaptive rule. The application of this multiobjective approach could be extended to multispecialty clinics colocated in the same waiting hall, sharing similar appointment periods and some common resources.

Several case studies on specialist outpatient clinics shed light on the development of this work. A detailed study of an ophthalmic specialist outpatient clinic was conducted in Singapore National Eye Centre [16]. The classification of the major patient groups (new and follow-up), the existence of pre- and post-consultation procedures, and the complex patient flow sequences are similar to those in this study. Four improvement strategies were proposed, including a new technology (dilation-free eye examination) and changing the appointment time slots (the duration and the ratio of slots between two patient groups) in an individual appointment system. Changing the appointment time slots was implemented and has reduced the average patient turnaround time (system time). Sharing some similarities with their work, we further target at improving the resource allocation in coordination with patient scheduling. In another study of eye outpatient clinics in a UK hospital [17], an initial qualitative approach was recommended, followed by a series of simple (or sometimes more sophisticated) quantitative models depending on the circumstances. Both long-term and daily operational strategies were proposed. From one of the authors' earlier work at an ophthalmology clinic of a public hospital, a simulation study on operational improvement [1] and a deterministic block appointment scheduling heuristic [2] were presented. The current work is an extension of [1, 2] by including the tactical decisions of resource allocation and the stochastic factors in the environment.

To summarize, this article addresses the following questions for a multiphase and multiserver queuing system with stochastic factors in order to optimize the (weighted average) objectives of patient waiting time, resource overtime, and waiting room congestion:

- Service discipline: When a clinical staff member is available, how is one of the patients waiting for treatment selected?
- Block appointment schedule: How does one decide on a block appointment schedule for a given number of patients, categorized by patient classes?
- Resource allocation: How does one allocate resources to tasks when each resource unit has one or more skills in handling different tasks in the system?

Having multiple skills will improve resource efficiency and flexibility in task allocation. Such *resource flexibility* will also contribute to the long-term development of continuity of care emphasized in healthcare. Continuity of care refers to practitioners handling their own set of patients, thus allowing them to be responsive to an individual patient's changing needs. It has been proposed as one of the strategy in enhancing primary care [18] as it will impact on the long-term health condition of patients. However, in local publicly funded specialty clinics, such practice has not been implemented yet. Patients are simply assigned to an available clinician (doctor, nurse) in every procedure. If resources were specialized in a single skill, there will be a trade-off in accessibility of healthcare providers when implementing continuity of care. In a community midwifery problem where midwives travel to the clients' home locations to provide postnatal care, the trade-offs between travel time and continuity of care were studied [19]. Results indicate that allocating and routing of midwives to their own set of clients cannot be achieved perfectly, but a high degree of 70% is feasible with relatively small additional travel time. The main obstacles to perfect continuity of care are shift patterns and part-time working of staff. It is suggested that with a reasonable flexibility in the schedule of home visits, a higher degree of continuity of care is achievable. This study has potential relevance to the current work in analysing the integration of service discipline, appointment schedule, and resource allocation. When continuity of care is to be introduced, having multiple skills will enable resources to serve a larger set of clients, thereby improving efficiency. Besides, there are resources and procedures still commonly shared by all patients. Similar to [19], the current work can serve as a base model to examine the trade-offs for implementing perfect or high degree of continuity of care.

**Figure 1:**
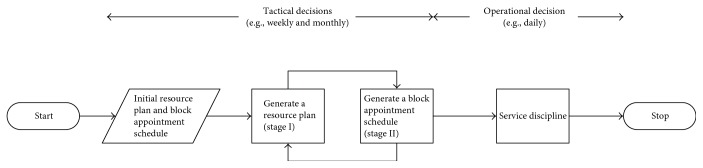
Overview of the integrated resource allocation and appointment scheduling problem.

Figure 1 shows a graphical representation of the approach with the time frame of the three decisions. The resource availability is known at a tactical level, say weeks or months before the appointment day. The daily appointment quota for each specialist clinic is usually set by considering various factors, including the service demand, manpower available, and capacity of physical facilities. Thus, the daily quota by a patient class is also available at the tactical level. The tactical decisions in the approach include finding an improved resource plan (stage I) and block appointment schedule (stage II) iteratively based on their initial configuration. At the daily operational level, the staff can decide on the service discipline. This is also included as an offline procedure in the appointment scheduling stage to evaluate the resulting performances. In addition to a proposed adaptive patient selection rule as service discipline, a number of well-known priority rules are applied for comparison. A two-stage algorithm in this article is defined as the application of both stages I and II (stage I + II) including a patient selection rule. All methods are compared with a base scenario defined by an initial resource plan and block appointment schedule with the first-come-first-served (FCFS) rule.

### 1.1. Research Questions

The methodologies to be analysed are classified in Table 1.

The following research questions are to be investigated in the experiments based on the setting and parameters from an ophthalmology clinic in earlier studies [1, 2]:

- How much improvement over the base scenario can be obtained from the different methods?
- Which algorithm performs better than the others?
- Is it sufficient to find a good resource configuration plan (stage I)? What is the additional benefit of appointment scheduling (stage II) over the integrated strategy (stage I + II)?
- What is the impact of resource flexibility?

The rationale behind research question (iii) is that under certain circumstances, such as equity concerns, operational constraints, and incomplete patient information, it may not be possible to optimize appointment scheduling. Question (iii) is explored by comparing the benefits of using both strategies together (stage I + II) over resource allocation without appointment scheduling (stage I only).

### 1.2. Contribution

To the best of our knowledge, there is little healthcare research aimed at integrating both resource allocation and patient scheduling decisions for tactical and operational planning. To solve problems of a realistic size with hundreds of outpatients scheduled for a half-day session requiring multiple procedures and resources, an iterative two-stage simulation-based optimization approach is proposed. The algorithm design is novel in its adaptive solution approach without requiring adding resources. If the patient flow statistics could be tracked in the system dynamically, the problem parameters can be updated periodically to automate the solutions for decision support.

This article is organized as follows.  Section 2 introduces the integrated problem. The decomposition of the integrated problem into two stages of the simulation-based heuristic follows in Section 3. The design of computational experiments and the methods for comparison are presented in Section 4. The results, discussion, and model limitations are given in Section 5. Finally, the conclusions and insights are drawn in Section 6.

## 2. Problem Description

The model assumptions of the integrated problem are derived from early studies of an ophthalmology clinic [1, 2] but can be generalized to multiphase and multiserver queuing systems. Assumptions (i)–(xi) mainly focus on patients and appointment scheduling, whereas the remaining assumptions (xii)–(xvii) focus on resources and allocation.

### 2.1. Model Assumptions

- A total of *Q* patients are scheduled for the appointment session.
- Each patient is categorized into exactly one of *M* patient classes with given class proportions. Patients in a class may be diverted to one or more treatment paths depending on their health conditions. Each path corresponds to a defined series of procedures treated by qualified resource units.
- A treatment procedure can be performed by any unit in the qualified resource group. The procedure operates on either a single patient or a (continuous) batch of patients.
- The start time of a treatment procedure on a patient must satisfy both the precedence relationship of the patient's treatment sequence (allowing movement time and record handling time between successive procedures) and the resource unit's completion time for the previous patient.
- The appointment session of duration *T* is divided into *K* time blocks. The first *K* − 1 time blocks have equal length (but equal length is not always necessary), whereas the last block behaves like a large time buffer until the end of the session.
- Each patient is scheduled to arrive at the start of exactly one of the *K* time blocks, though the actual arrival times may be different.
- The congestion level (or queue length) is measured by the number of patients and their accompanying visitors waiting for a procedure in the clinic waiting area. Patients and visitors queuing or attending procedures outside the clinic waiting area (e.g., registration and appointment booking) will not be included in the congestion headcount.
- Visitors accompanying patients will follow them throughout their outpatient service process (including waiting and going into the treatment room).
- The stochastic factors considered include patient punctuality, earliness/tardiness regarding the appointment time, a patient in a class being diverted to different treatment paths, procedure time, and the number of visitors accompanying each patient.
- Given that appointment reminders are issued by hospital staff and that the demand for outpatient services (in public hospitals) is high, no-shows either are not considered or have been accounted for when deciding on the appointment quota (*Q*) in assumption (i).
- To avoid a further increase in staff workload (observed in some public hospitals), an overbooking strategy is not adopted nor has it been accounted for in assumption (i).
- The total number of resource units in the resource set (*ℜ*) and the skill set of the individual resource units are given at the tactical level and remain constant during the planning horizon.
- Each resource unit is assigned to a single treatment procedure or a combined set throughout the appointment session (e.g., registration and appointment booking are often combined and assigned to one or more resource units).
- A resource unit is fully assigned to perform a batch procedure throughout the appointment session.
- Resource units assigned to perform a treatment procedure (or a combined set) are considered identical and have the same service rate.
- Each procedure should be assigned to at least one resource unit.
- Each resource unit has the flexibility to select any patient waiting for his or her next treatment.

### 2.2. Multiple Objectives

The integrated problem has the multiple objectives of minimizing the average patient waiting time (*Z*_1_), the average resource overtime (*Z*_2_), and the average congestion level (*Z*_3_) expressed as a weighted function in

(1) $$\begin{array}{c} \mathrm{Min}.\;Z=\overset{3}{\underset{h=1}{\sum }}w_{h}\cdot Z_{h}, \end{array}$$

where *w*_*h*_ represents the weight or relative importance of objective *h* ( = 1, 2, 3) decided by the user. A similar weighted performance measure has been adopted in [7] for the average system time and average clinic overtime. On the basis of the observed practice [1], early arriving patients are allowed to start registration in order for the service to be people centered and to avoid resource idle time. Accordingly, the start of patient waiting time is defined by the actual patient arrival time (stochastic) or the beginning time of the appointment session, whichever is later. The overtime of a resource unit is the excess working time beyond the session duration (*T*), if any. The average overtime per resource unit (*Z*_2_) is the sum of overtimes divided by the total number of resource units. The average congestion objective (*Z*_3_) only considers patients (and visitors) waiting for treatment procedures in the clinic waiting area as some procedures are performed externally. This is estimated by Little's law for the average queue length (*L*_*q*_ = *λ* · *W*_*q*_) [20]. It can be expressed alternatively as the sum of the time spent on waiting in a queue for all arrivals, divided by the duration of the observed period. The arrivals include patients and visitors (∑_*j*=1_^*Q*^(1 + *ν*_*j*_)) observed during the session duration (*T*), where *v_j_* is the number of visitors accompanying patient *j* ( = 1,…, *Q*).

### 2.3. Model Decisions

The three tactical and operational decisions (Figure 1()) optimizing the weighted objective function (1) are defined in the following. Their integrated and separate effects will be analysed in the computational experiments for investigating the research questions (Section 1.1).

(D1) Resource configuration plan (R): Assignment of each resource unit (*r* ∈ *ℜ*) to one of its qualified procedures (single or combined set) for the entire appointment session
(D2) Block appointment schedule (A): The number of patients (*Q*_*ik*_) from a patient class (*i* = 1,…, *M*) scheduled for the start of an appointment time block (*k* = 1,…, *K*), where (∑_*i*=1_^*M*^∑_*k*=1_^*K*^*Q*_*ik*_) = *Q*.
(D3) Service discipline (S): Selection among the waiting patients of the next patient for treatment, whenever a resource unit is free.

The manpower available by a resource group is known at the tactical level. Even at the operational level, the assignment of resource units to qualified procedures (D1) can be improved for a given appointment schedule or by considering all three decisions simultaneously. The block appointment schedule (D2) decided at the tactical level can serve as a reservation list for making future appointments. The service discipline (D3) at the operational level will utilize the system status information, including the resource units and their waiting patients.

### 2.4. System Constraints

The system constraints in a multiphase and multiserver system are typically related to managing supply, demand, and the flow sequence. These include the allocation of each unit of different resources to qualified procedures (assumptions (xiii) and (xiv)) and the staff requirement of each procedure (assumption (xvi)). Assigning patients to time blocks ensures that each patient will be scheduled to exactly one appointment time block (assumption (vi)). Conversely, the sum of patients assigned to each time block must not fall below a minimum limit, to avoid resource idle time and possible overloading of other time blocks. *Patient arrival times* are stochastic, and patients may arrive earlier or later than the scheduled appointment time, but not earlier than the facility opening time. The *procedure start tim*e for each patient must not be earlier than the available starting time of the assigned resource unit(s). The *precedence constraints* ensure that a patient's procedure can only start when the preceding procedure has been completed, allowing a time gap for patient movement and for staff to handle records. Similarly, time constraints for a resource unit apply to every consecutive pair of patients being treated sequentially. The *capacity constraint* for the continuous batch procedure restricts the number of patients (and accompanied visitors) processed at any time so that it does not exceed a maximum limit. (An example is a video session for educating day surgery patients and accompanying visitors. The video is played continuously and repeatedly, and the maximum limit is the room capacity.)

Regarding the problem complexity, a special case of the deterministic problem is the flow shop scheduling problem having a fixed resource plan with identical treatment sequences for all patients and the single objective of minimizing mean system time (or mean waiting time plus a constant). This special case has been proved to be NP-complete [21]. Consequently, a heuristic approach applying simulation to handle the uncertainties is proposed for the current problem.

## 3. Methodology

To tackle this complex integrated problem that involves stochastic factors, a novel two-stage simulation-based heuristic is proposed. The resource allocation (stage I) is the high-level problem, and the block appointment scheduling including the service discipline (stage II) is the secondary problem, or subproblem. The methodology used is an iterative heuristic optimization algorithm with probabilistic search and memory structures. The details of each stage will be explained in a top-down manner.

### 3.1. Resource Allocation Problem (Stage I)

The main decision is the resource configuration plan (D1 in Section 2.3), defined by the number of compatible resource units assigned to perform each (single or combined) procedure. This stage is activated whenever stage II termination condition is met. The waiting time statistics from the most recent appointment schedule recorded at the end of stage II are used to reallocate resource units among their qualified procedures to improve the weighted objective (1). Despite not having a convergence proof of optimality, this approach of using waiting time statistics for resource allocation is patient centered. It is inspired by the demand diversion strategy of motivating specialty patients to visit hospitals in clusters with short waiting times [22]. Assumption (xii) is crucial for this stage as resource flexibility (skill set) determines the degree to which resource units can be reallocated.

#### 3.1.1. Reduce Deviation in Average Waiting Time (Stage I)

**Figure 2:**
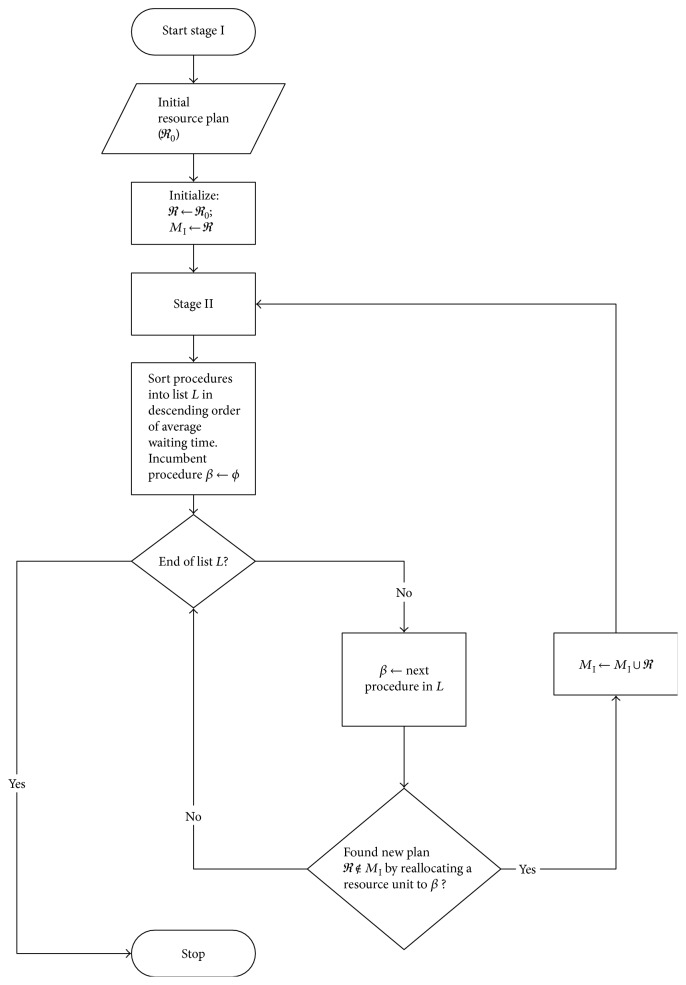
Stage I: Find a new resource plan (*ℜ*) to reduce deviation in average waiting time among procedures.

The objective of the high-level resource allocation problem is to reduce the deviation in the average waiting time among procedures by reallocating resource units (Figure 2()). This helps alleviate the workload of busy servers. A memory of the resource plans examined (denoted by *M*_I_) is maintained to avoid repetition. For this complex problem with the three layers of discrete decisions (Section 2.3), achieving computational efficiency is also important. A greedy resource allocation approach offers a quick, improved solution, but with no guarantee of solution quality owing to its heuristic nature. The concept is to identify the busiest procedure, denoted by *β*, with the largest average waiting time, and to reallocate a compatible resource unit from another procedure with a smaller average waiting time to *β*. This is implemented by sorting the average waiting time statistics by procedure in descending order, as calculated from the recent set of replications of the simulation (stage II). The associated procedures are placed in a list called L. The first procedure in L is considered to be the busy procedure (*β*), and a compatible resource unit from another procedure (in the reverse order of list L) will be identified for reallocation to *β.* If the resulting resource plan *ℜ* is new and feasible (i.e., at least one unit is assigned to each procedure), *ℜ* will be recorded in stage I memory (*M*_I_). Otherwise, the process will repeat using the next procedure in L. Once a new feasible plan *ℜ* is found, stage II (appointment scheduling) starts again with the recent appointment schedule (*π*), and the stage II memory (denoted by *M*_II_) will be refreshed as *ℜ* is new and unique. Eventually, when no new feasible plan *ℜ* can be found, stage I will terminate, and the entire algorithm will end. The best-recorded resource plan is the one associated with the best appointment schedule (*π*_best_) found in stage II.

### 3.2. Block Appointment Scheduling Problem (Stage II)

**Figure 3:**
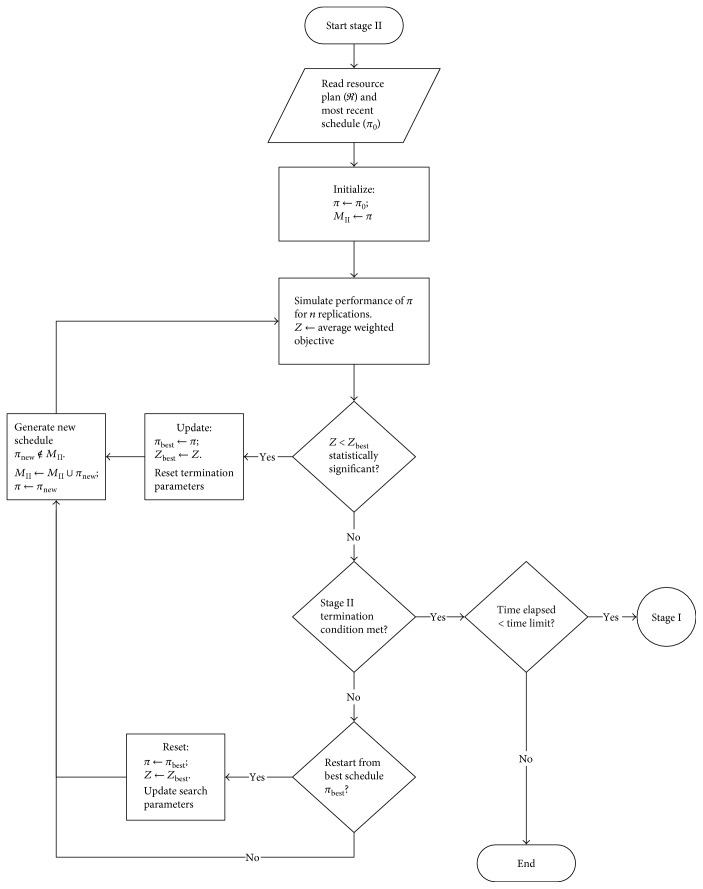
Stage II: Simulation-based heuristic for block appointment scheduling.

In past studies, the appointment scheduling problem mostly focused on a single clinic. With certain modifications, the proposed method could be extended to multiple clinics sharing some common resources and waiting areas. The tactical decision of block appointment schedule (D2 in Section 2.3) is searched heuristically for a given resource allocation plan (*ℜ* in stage I). This stage includes the service discipline (D3 in Section 2.3) as an offline operational procedure for performance evaluation and comparison. The solution method is an extended development of an adaptive scheduling heuristic for the deterministic problem [2]. The new development in this stage includes three aspects: improving the patient selection rule on the basis of the dynamic status information; enhancing the new schedule generation mechanism; and incorporating simulation into the optimization framework to handle uncertainties. The design logic of stage II is shown in Figure 3().

#### 3.2.1. Evaluating Schedule Performance

Starting with a given resource allocation plan from stage I, the performance of the most recent block appointment schedule (*π*_0_), defined by {*Q*_*ik*_, *i* = 1,…, *M*, *k* = 1,…, *K*}, will be forecasted by simulating the stochastic factors using discrete event simulation. Each schedule will be run for a given replications to ensure the margin of error $\left(=t_{\alpha /2,n-1}\cdot \left(s/\sqrt{n}\right)\right)$ in estimating the weighted objective value is not more than a predetermined limit 100*ε*% from the sample average (*Z*) at a 100 (1−*α*)% level of confidence. (*s* is the sample standard deviation of the weighted objective from the *n* replications of the simulation.) As in stage I, a memory of block appointment schedules generated under the current resource plan, denoted by *M*_II_, will be retained to avoid repeating the same schedule. At the end of stage II, the last schedule and its performance statistics will be used to generate a new resource allocation plan (stage I). Except for this last schedule (to be used as the starting schedule in the next iteration of stage II) and the best overall schedule (*π*_best_), the other schedules in the memory of *M*_II_ will be cleared.

#### 3.2.2. Patient Selection Rule (or Service Discipline)

This operational decision is included as an offline procedure in stage II together with the appointment schedule to test its impact on the objective. In another study of an ophthalmology clinic [16], apart from the commonly used first-come-first-served (FCFS) rule, selection by appointment time order is also adopted in certain procedures, such as evaluation and consultation. The benefit of flexible patient selection is explored here under assumption (xvii). For the current multiphase and multiserver complex network with a large number of patients per session and frequent updates of system status, the adaptive rule proposed in (2) calculates the immediate impact of a selected patient on the objective function (1). It suggests selecting the patient (*j*^∗^) from the set of waiting patients, denoted by *Ω*, that has the least undesirable impact, Δ_*Z*_(*j*^∗^), on the (weighted) objective value:

(2) $$\begin{array}{c} \Delta_{Z}\left(j\right)=\left(\frac{w_{1}\cdot {count}_{p}}{Q}+\frac{w_{3}\cdot \left({count}_{I}–1–v_{j}\right)}{T}\right)\cdot E\left(t_{j,l_{j}+1}\right)-\frac{w_{2}}{\left|R\right|}\cdot \overset{n_{j}}{\underset{l=l_{j}+1}{\sum }}E\left(t_{jl}\right),\;j\in \Omega , \end{array}$$

(3) $$\begin{array}{c} \Delta_{Z}\left(j^{*}\right)=\underset{j\in \Omega }{\min}\left\{\Delta_{Z}\left(j\right)\right\}. \end{array}$$

Consider an available resource unit and one of its waiting patients *j* ∈ *Ω*. Let *l*_*j*_ and *l*_*j*_ + 1 be the recently completed and current procedure for patient *j*, respectively. Apart from patient *j*, suppose there are count_p_ ( = |*Ω*| − 1) waiting patients that can be selected by the same resource unit. If the current procedure is performed inside the clinic, let count_I_ be the sum of waiting patients and accompanying visitors, and 0 otherwise. The impact of selecting patient *j* on each of the three objectives is explained separately as follows:

- The first term in (2) represents the estimated impact on the objective of average patient waiting time (with weight *w*_1_) when the count_p_ patients will each be delayed by patient *j* with expected treatment time *E*(*t*_*j*,*l*_*j*_+1_). The total delay is averaged over the total number of patients (*Q*), resulting in an overall expression of *w*_1_ · count_p_ · *E*(*t*_*j*,*l*_*j*_+1_)/*Q*.
- Similarly, for the objective of average congestion in the clinic waiting area (with weight *w*_3_), selecting patient *j* will cause a delay of *E*(*t*_*j*,*l*_*j*_+1_) for each other waiting patient and accompanying visitors. These are then converted into the congestion measure. With a total of count_I_ patients and visitors currently waiting for the resource unit, count_I_ − 1 − *v*_*j*_ will remain if patient *j* is selected where *v*_*j*_ is the number of accompanying visitor(s). The impact on the congestion objective is estimated by using Little's law (*L*_*q*_ = *λ* · *W*_*q*_) [20], by dividing the sum of the waiting times in the queue, (count_*T*_–1–*v*_*j*_) · *E*(*t*_*j*,*l*_*j*_+1_) by the session duration (*T*). This results in the second weighted expression in (2).
- The last term in (2) represents the estimated impact on the objective of average resource overtime (with weight *w*_2_) over all resource units. Resource overtime is *related* to the remaining treatment time for patients with unfinished treatments. For the current resource unit, all the waiting patients (set *Ω*) require an expected total remaining treatment time of ∑_*i*∈*Ω*_∑_*l*=*l*_*i*_+1_^*n*_*i*_^*E*(*t*_*il*_), which is a constant at this time point. If patient *j* is selected, the expected remaining sum of treatment times (excluding *j*) per resource unit would be (∑_*i*∈*Ω*_∑_*l*=*l*_*i*_+1_^*n*_*i*_^*E*(*t*_*il*_) − ∑_*l*=*l*_*j*_+1_^*n*_*j*_^*E*(*t*_*jl*_))/|*R*|, where |*R*| is the total number of resource units. Ignoring the constant term ∑_*i*∈*Ω*_∑_*l*=*l*_*i*_+1_^*n*_*i*_^*E*(*t*_*il*_) gives the weighted expression in the last term of (2).

Equation (2) represents a rule governing the trade-off between the immediate treatment time and the remaining treatment time. (If the system is entirely patient centered, that is, *w*_2_ = 0, (2) and (3) become the shortest processing time rule.) Conversely, if the average resource overtime dominates, that is, *w*_2_ > 0, *w*_1_ = *w*_3_ = 0, (2) and (3) would select the patient with the longest remaining treatment time, and this is known as the critical path rule.) Equation (3) chooses the ideal patient (*j*^∗^) with the least undesirable impact expressed by (2). An implication of this adaptive rule is that a patient with a short immediate treatment time and a long remaining treatment time is always preferred over different weights. When ties occur in choosing the ideal patient (*j*^∗^) from (3), the FCFS rule can also be applied to ensure fairness.

#### 3.2.3. Generating a New Schedule

The performance of every incumbent appointment schedule (*π*) will be evaluated by running *n* replications of the simulation. (*n* is chosen such that the margin of error in estimating the true mean objective value is within 100*ε*% of the sample mean value.) The procedure used to generate a new schedule from the current schedule *π* employs a probabilistic greedy approach. The rationale behind it is to improve the objective function by rescheduling a pool of patients from time blocks having a greater *undesirable* impact on the objective to time blocks having less of an impact. The impact of a patient class is the sum of the weighted objective values from all its patients. If the simulation results reveal that a certain patient class would have a large impact on (i.e., contribute a larger value to) the objective function of the current schedule, patients in such a class would be given a larger probability of being selected for rescheduling.

First, a procedure that has caused a large impact is selected *probabilistically*. Next, a related patient class is identified, and one of its patients is rescheduled from a time block that has a greater impact to another time block that has less impact. The selection of a procedure, related patient class, and time blocks (of removing and reinserting patients) are based on probability distributions constructed from the performance statistics recorded in the recent *n* replications. The target characteristics (e.g., having a large impact on the objective) are given a greater probability of selection by construction. These procedures for selecting a patient will be repeated until a pool of *p*_size_ patients has been rescheduled to create a new schedule (*π*_new_) not recorded in memory *M*_II_. (If *π*_new_ has occurred before, another pool of *p*_size_ patients will be selected for rescheduling.) *π*_new_ is then added to the memory *M*_II_ to replace the incumbent schedule (*π*), and its performance is evaluated by simulation.

#### 3.2.4. Testing for an Improved Schedule and Termination (Stage II)

Whenever an incumbent appointment schedule (*π*) is evaluated by the simulation, the average objective value *Z* over the *n* replications will be compared with the best-recorded objective value *Z*_best_ from the associated schedule *π*_best_. An improved schedule is tested by the research hypothesis *Z* < *Z*_best_ (versus the null hypothesis *Z* ≥ *Z*_best_) at a predetermined 100*γ*% significance level. If the test is statistically significant, *Z*_best_ and *π*_best_ will be updated by *Z* and *π*, respectively. The algorithm design includes intensification and diversification search strategies. Whenever an improved schedule is identified, the maximum number of selected patients for rescheduling (*p*_max_size_) will increase, allowing the search time to extend before reaching the stage II termination condition. When restarting from the best schedule, the pool size parameter (*p*_size_) is increased by one to slightly enlarge the size of the neighbourhood for searching new schedules.

### 3.3. Base Scenario

The base scenario is a resource plan and a block appointment schedule collected at an ophthalmology specialist outpatient clinic in a public hospital [1, 2] while assuming the first-come-first-served (FCFS) patient selection rule. They are also adopted as the initial resource plan (*ℜ*_0_ in stage I) and the initial schedule (*π*_0_ in the first iteration of stage II) in the proposed method.

### 3.4. Priority Rules for Comparison

In addition to the proposed patient selection rule in (2) and (3) and the FCFS rule in the base scenario, a number of well-known priority rules are applied for comparison. Each of these rules benefits one or more of the three objectives (1). They are as follows: shortest processing time first (SPT), largest number of successors (LNS), critical path (CP), and shortest queue at the next operation (SQNO). (A review of the priority rules can be found in [23].) In appointment scheduling literature, scheduling patients with low variance first is known to be effective in balancing between patient waiting time and server idle time [24]. We apply a closely related rule, the low range (LR) rule, which is to select the waiting patient with the smallest range in treatment time as the range information is available from the earlier study [1] for the current experiments. Each of these priority rules is combined with the stage I or two-stage algorithm (Sections 3.1 and 3.2), as an alternative of (2) and (3), to explore further the impact of integrating strategies.

## 4. Computational Experiments

The classification of parameters in outpatient scheduling problems and solution methods can be found in a survey [25]. In a local case study [1, 2] related to this work, all medical procedures are performed inside the clinic while the registration and appointment booking counters are located outside the clinic. The problem is generalized to a multiphase and multiserver queuing system sharing some common resources or waiting area with multiple conflicting objectives.

### 4.1. Operating Parameters

Table 2 lists the parameters representative for an outpatient setting.  Table 3 shows their values based on data collected from the case study [1, 2]. The number of appointments at the time of the study was 200 for a session of 4.5 hours. To account for growth in demand, a 25% increase is assumed here with the original staff size and operating conditions. The distribution of the appointments among the time blocks is maintained in the same proportion by assumption. To avoid resource idle time and overloading in other time blocks, a minimum number of appointments per time block are imposed here. The minimum limit is assumed to depend on the total number of appointments (*Q*) and number of time blocks (*K*) by adopting a simple function ⌊*Q*/*K*/3⌋ = 6. The data collected on earliness and tardiness (Table 3) revealed the same probability of early arrival for different patient classes. Patient punctuality is simulated in the experiments using the empirical distribution of data collected in Table 3. Resources are grouped by doctors, nurses, and equipment (room). Doctors start later than the start time of the appointment session (assumed time 0) due to assigned duties before the outpatient session, and patients also have other preconsultation procedures. Nurses are assigned to different procedures requiring different skills and experiences (Table 4). The resource allocation plan was collected from the original operating conditions [1, 2] while their additional skills are assumed and explained in the next section. The procedure information and related parameters (Table 5) were provided by the hospital management during the case study.

### 4.2. Resource Flexibility Scenario

When resource units possess multiple skills, reallocating such units among their qualified procedures will improve system performances for a given appointment schedule. Reallocation is feasible at the operational level as staff on duty and the appointment schedule are known at least a day before the appointment session. Sharing of resource (nurse with multiple specialisms) is observed in practice even between different specialist outpatient clinics when manpower is scarce in one clinic at the operational level.

To examine the impact of resource flexibility on performance, a scenario of the resource skill sets is created, representing some degree of flexibility in reallocation. In some clinics, such as oncology clinics, a high degree of specialized skills is required, as oncologists are trained to specialize in different cancer types. A medium-term resource allocation tool would be helpful for training/recruiting staff with the right set of skills [14]. The rationale behind the currently created scenario is that doctors would concentrate on consultation; experienced staff (senior nurses) would handle procedures requiring higher degrees of judgement (e.g., nurse assessment), and some staff can be reallocated to more routine procedures, depending on demand during the session. Junior staff would handle clerical procedures (e.g., registration and appointment booking) for different patient classes. The created scenario in Table 6 represents a resource set with multiple skills offering some degree of flexibility for reallocation. It is assumed that clerical procedures (registration and appointment booking, that is, V + VI) for different patient classes can be handled by the same group of nurses or clerical staff (N13–N15). All staff (N3–N9) who can perform visual acuity/eye examination (procedure III) are assumed to be able to measure eye pressure/apply eye drops (procedure IV). A few of them (N3) can also perform assessment tasks requiring more experience (procedure II).

### 4.3. Objective Weights

A numerical approach is adopted to examine the solution quality of the two-stage simulation-based heuristic over a tested range of objective weights (*w*_1_, *w*_2_, and *w*_3_ in (1)). By standardizing the weight of the average patient waiting time objective to 1 (i.e., *w*_1_ = 1), the ratio of importance (*w*_2_ : *w*_1_) between average resource overtime (*Z*_2_) and average patient waiting time (*Z*_1_) is tested over the range from 0 to 10, specifically, *w*_2_ = {0, 0.1, 0.2, 0.4, 0.6, 0.8, 0.9, 1, 2, 4, 6, 8, 10} versus *w*_1_ = 1. Similarly, the ratio of importance (*w*_3_ : *w*_1_) between the average congestion level (*Z*_3_) and average patient waiting time (*Z*_1_) is tested simultaneously over the range from 1/3 to 3, specifically, *w*_3_ = {1/3, 1/2, 1, 2, 3} versus *w*_1_ = 1. Overall, this results in 1 × 13 × 5 = 65 instances of different weights (*w*_1_, *w*_2_, *w*_3_) being run for every method.

### 4.4. Labelling of Instances

The 65 instances are divided into 5 groups with 13 instances per group. Group *g* contains instances labelled as 13(*g* − 1) + 1 to 13*g*, where *g* = 1, 2,…, 5. To facilitate comparison of methods in Section 5, each group consists of 13 pairs of (*w*_1_, *w*_3_) with the same values while *w*_2_ increases within the group over the stated range (Section 4.3). Between the groups, the weight on the congestion objective (*w*_3_) increases.

### 4.5. Algorithm Parameters

The algorithm parameters (Table 7) for the two-stage simulation-based heuristic are chosen after running the initial experiments. The parameters in the block appointment scheduling problem (stage II) are modified from those in the deterministic scheduling problem [2] to strike a balance between exploring a sufficiently large number of resource plans (stage I) and rescheduling patients (stage II) within the maximum time limit. The resource plans are explored systematically based on greedy reallocation, but the search is not exhaustive. To generate a new schedule in stage II, a pool of patients of initial size *p*_0_ ( = 20%×*Q*/iter_max_) is selected from the incumbent schedule (*π*) for rescheduling. (This allows a minimum proportion, 20%, of all *Q* patients in *π* to be rescheduled in every execution of stage II.) All algorithms have been coded in Microsoft Visual Basic .NET 2010 version and are performed on an Intel(R) Xeon(R) CPU E31270, 3.4 GHz processor. The maximum time limit (*t*_lim_) allowed for each algorithm on each test instance is 7200 CPU seconds.

## 5. Results and Discussion

To investigate the research questions (i)–(iv) in Section 1.1, the two-stage simulation-based heuristic is compared with the base scenario (Section 3.3) and the integrated strategies with the priority rules (Sections 3.2.2 and 3.4). Results over the 65 instances, and insights drawn, are given as follows.

### 5.1. Analysis of Research Questions

#### 5.1.1. Improvement over the Base Scenario

**Figure 4:**
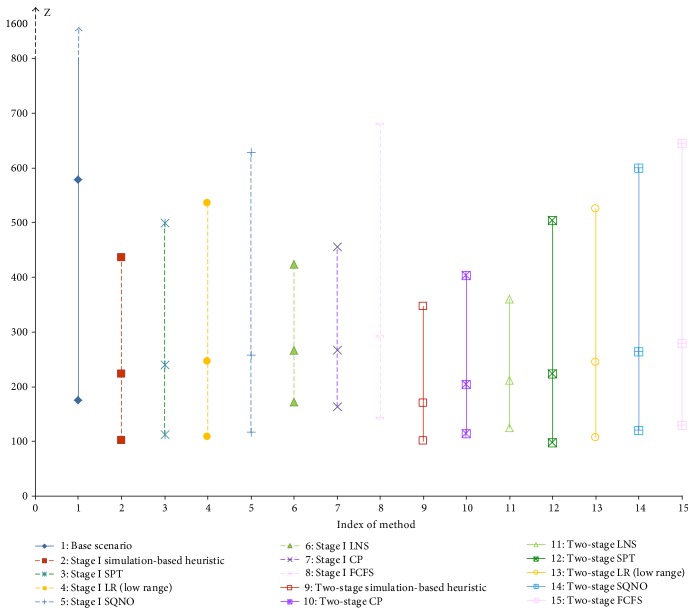
Performance comparison of stage I and two-stage algorithms with the base scenario on the (minimum, average, and maximum) objective.

The base scenario is compared with the two-stage algorithms and stage I algorithms in Figure 4. Each vertical line shows the minimum, average, and maximum objective value of each algorithm over the 65 instances. In Figure 4 and in every instance, the base scenario representing the initial plan can be improved by any integrated strategy. Simply finding a better resource plan (stage I) while adhering to the FCFS rule can result in an average improvement of 43%. If the patient selection rule in (2) and (3) is used (stage I simulation-based heuristic), the largest average improvement of 53% is recorded. Further incorporating the appointment scheduling strategy can lead to more benefits at the expense of computational effort and time.

In terms of computational time, the base scenario takes negligible time in the simulation. The stage I algorithms take between 120 CPU seconds for priority rules and 1400 CPU seconds for the simulation-based heuristic. The two-stage algorithms would take between 3000 CPU seconds to the maximum time limit of 7200 CPU seconds for the priority rules and the simulation-based heuristic, respectively.

#### 5.1.2. Comparison between Algorithms

**Figure 5:**
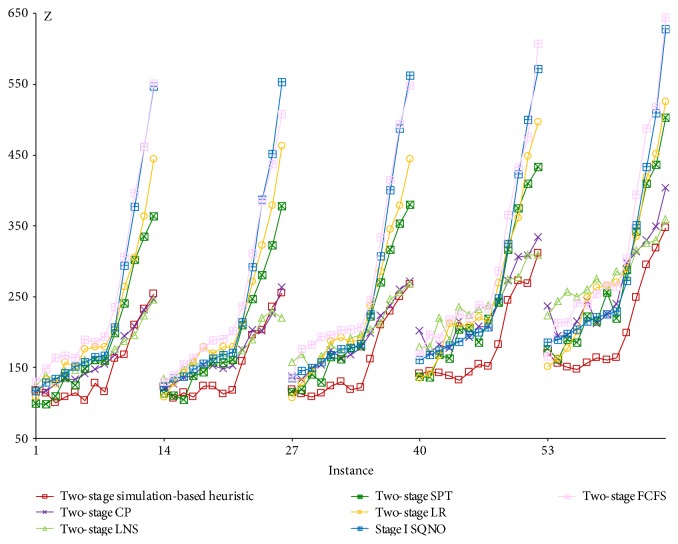
Comparing the algorithms with their best integrated strategy.

**Figure 6:**
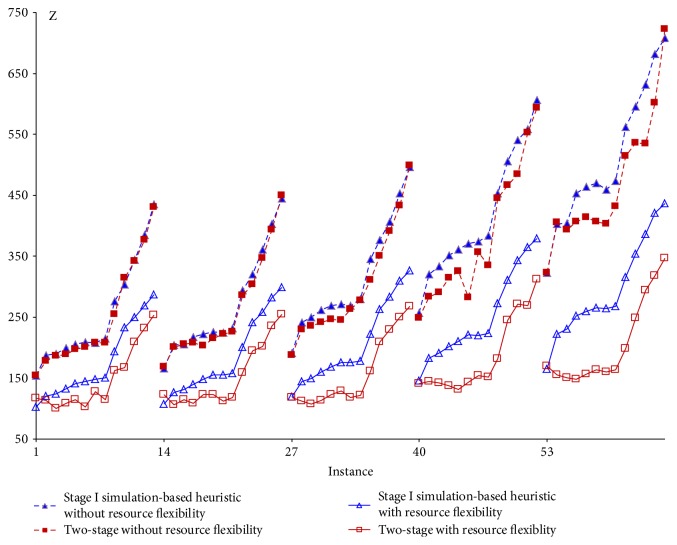
Comparing the stage I/two-stage simulation-based heuristic with/without resource flexibility.

**Figure 7:**
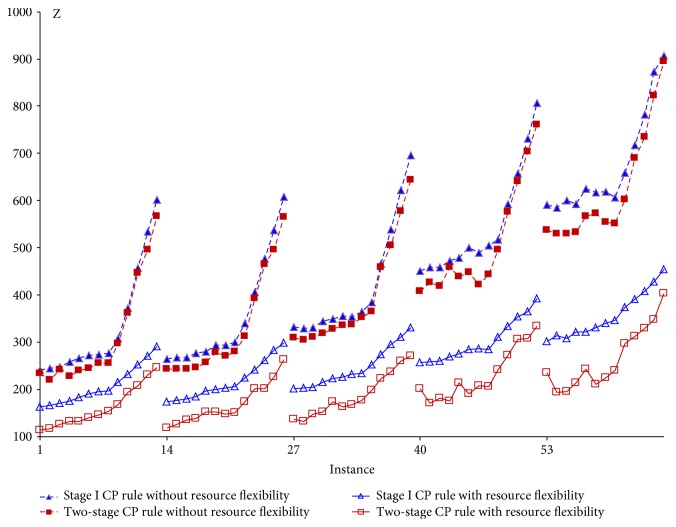
Comparing the stage I/two-stage critical path (CP) rule with/without resource flexibility.

**Figure 8:**
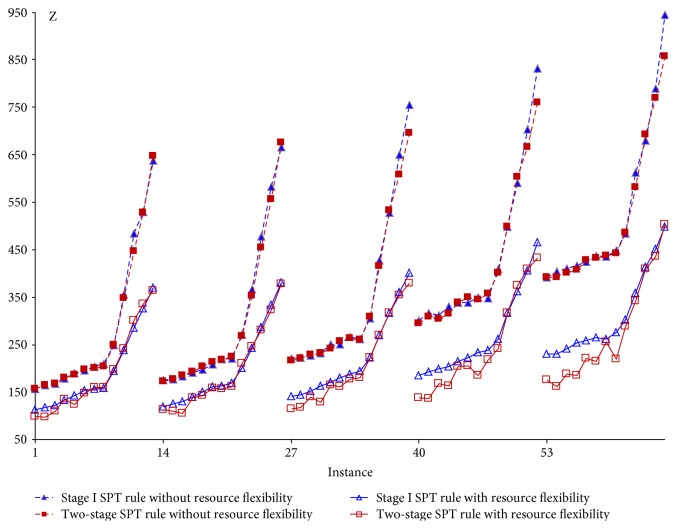
Comparing the stage I/two-stage shortest-processing time first (SPT) rule with/without resource flexibility.

As stated in Section 4.4, the 65 instances are divided into 5 groups with each group indicated by its first instance in Figures 5, 6, 7, and 8. Within each group, the weight of the resource overtime objective (*w*_2_) increases from 0 to 10 while the other two objective weights are kept constant. From group 1 to group 5, the weight of the congestion objective (*w*_3_) increases from 1/3 to 3.

To find the best overall algorithm for the specialty clinic under study, the best integrated strategy for each patient selection rule ((2) to (3) and Section 3.4) is selected for comparison in Figure 5(). In addition, one-tail paired *t*-test is applied for pairwise comparison at the 5% significance level as described in Section 5.1.3 below. From Figure 5() and the statistical results, the high performers on the objective are listed in the following priority order: two-stage simulation-based heuristic, two-stage CP, two-stage LNS, two-stage SPT, two-stage LR, stage I SQNO, and two-stage FCFS. The two-stage simulation-based heuristic using (2) to (3) is more flexible over different weights and outperforms all the others. As expected, when resource overtime is more important (large *w*_2_), the two rules CP and LNS perform better than SPT and vice versa.

#### 5.1.3. Impact of the Resource Allocation Strategy (Stage I Only) with/without Appointment Scheduling

The integrated strategy two-stage algorithm is compared with stage I only under each patient selection rule. The representative ones are depicted in Figures 6–8 showing their respective performance with and without resource flexibility. Figures 6–7 show that under two patient selection rules, (2) to (3) and CP, finding good appointment schedules, combined with resource allocation will result in significantly more benefit than applying resource allocation only. (Similar performance is observed in the LNS rule.) However, under the SPT rule (Figure 8), improvement from appointment scheduling is observed only for small values of the weight on resource overtime (*w*_2_). This implies the stage I SPT rule combined with appointment scheduling will give improvement when patient waiting time is considered more important than resource overtime, as well as when congestion is given more concern (large *w*_3_). For the remaining three patient selection rules (LR, FCFS, and SQNO), the stage I and two-stage algorithms do not show much difference in performances, implying the resource allocation strategy is sufficient.

More specifically, a one-tail paired *t*-test is applied to each pair of comparison at the 5% significance level. The two-stage algorithm with each patient selection rule performs better on the objective, except for the LR and SQNO rules. The LR rule shows no difference when integrating with either strategies while the SQNO rule can perform better with the stage I strategy.

#### 5.1.4. Impact of Resource Flexibility

If there were no resource flexibility as in Table 6, each resource unit has only a single skill which is the assigned procedure in the initial resource plan (Table 4). Each integrated strategy would then terminate at the end of this first resource plan (*ℜ*_0_) when stage I is executed once. The best-recorded objective at this point will be compared with the best found (*Z*_best_) when resource flexibility exists, that is, when stage I is executed multiple times. For the 7 patient selection rules, the stage I and two-stage algorithms with resource flexibility are each compared with their counterpart without resource flexibility. Figures 6–8 show the case for three patient selection rules. Significant difference between the objective values (*Z*) is observed in each pair of comparison. This implies creating resource flexibility has great impact regardless of the integrated strategy and/or patient selection rule adopted.

### 5.2. Choice of Objectives and Weights

For publicly funded specialist outpatient clinics which are often resource constrained, resource utilization including overtime is a typical concern. Emphasis on service quality has led to implementation of many measures by the hospital management to increase service capacity and manage waiting time. If congestion is to be controlled due to capacity concern, changing this objective to a constraint by imposing a bound (e.g., physical capacity limit) would be realistic. Then the only trade-off remaining is between resource overtime and patient waiting time. Publicly funded clinics typically serve a larger number of patients per session than the private ones. Reducing congestion helps mitigate the risk of infection. Naturally, there are other objectives that could be considered, such as maximizing preferences of staff, patients, and operational conditions. These could be treated as soft constraints, and penalty costs are imposed in the objective function when they are violated. A multicriteria operating theatre timetabling problem [26] demonstrates another example of many objectives. When there are more than two objectives, using a weighted function combining multiple objectives allows the trade-offs to be examined analytically. It is also easier to handle than the efficient frontier approach. When setting up appointment policies, the calibration of the ratio of the server completion time to expected customer waiting time is not an easy task [27]. Incorrect estimation of this ratio can lead to loss of utility of server and customer. The estimation of weights in [26] has shed light on these parameter values. The weights are also used to balance various objectives designed to smooth bed usage, give surgeons preference on time slots and repeated weekly assignments. It is suggested to use equal weights initially and then adjust them according to the changes desired in the resulting timetable. Similarly, in the proposed simulation-based heuristic with (2) and (3), it is suggested to start with equal objective weights. Taking advantage of the relatively short running time of the stage I algorithm, run the stage I simulation-based heuristic to adjust the weights until the objective values are desirable or no further improvement is observed. Then apply the incumbent set of weights to run the two-stage algorithm to further optimize the objectives. This approach is supported by the instances tested in this work.

### 5.3. Examining Multiple Objectives

After concluding the investigation of the research questions (i) to (iv) in Section 1.1, the two-stage simulation-based heuristic using (2) and (3) will be examined more closely in its solution quality. Optimizing a single (weighted) measure serves as a tool for deriving a good *balanced* solution over all objectives.  Table 8 shows the case where resource overtime is the most important and congestion is the least (*w*_1_ = 1, *w*_2_ = 10, *w*_3_ = 1/2) for the two best algorithms, two-stage simulation-based heuristic with (2) and (3), and two-stage with CP. The improvement from the first to the best solution is drastic for each algorithm. Both are far better than the base scenario.

To summarize, the improvement in the weighted objective (*Z*) when applying the appointment scheduling strategy to a resource plan is usually gradual, compared with the change between successive resource plans. Flexibility in reallocating resource units could lead to a drastic improvement if this process can be automated to find the right configuration plan (which is often not the most obvious one tested). A single/composite priority rule could achieve significantly better performance when combined with both resource allocation and appointment scheduling, or even only with resource allocation.

With only a single strategy, improvement could be limited by the initial conditions. The two-stage simulation-based heuristic using (2) and (3) is the best performer on average and is more flexible in handling the different weights of the conflicting objectives (Figure 5). Nevertheless, when staff overtime is the top priority, the integrated strategy with CP or with LNS can sometimes give better results. A small degree of resource flexibility targeting the bottleneck procedures can lead to a great improvement. This is analogous to the conclusion in [14] that operational efficiency can be improved by optimizing the oncologist specialization mix. The importance of matching not only the demand volume but also the request and resource types is also demonstrated in this study.

### 5.4. Discussion and Limitations of Study

This section discusses some limitations of this study followed by the response from the hospital clinic in our study.

Our method requires the data on patient class information, resource availability, and skill sets to be known in advance. Data collection could be facilitated by extracting the electronic health records of patients, staff roster, and personnel records. Information technology support could help automate this process.

On the variability of data, the empirical data collected from the specific clinic (Table 3) has been used in the simulation of parameters. In addition, a sample of 89 doctors' consultation times has been collected in the previous joint study [1]. They revealed a multimodal distribution with extreme values of 3 and 25 minutes. The stated range of consultation time from 5 to 10 minutes (Table 5) provided by the hospital has similar average and has captured 70% of the majority of collected values. Hence, it has been used in simulating the consultation time variable. Other procedure times (Table 5) all have small range of not more than 5 minutes. For simplicity, a uniform distribution has been assumed for each variable in Table 5. If data have larger variability, the standard error of the estimated objective function (*Z*) increases. This is observed in additional computational experiments when the range of procedure duration (Table 5) is doubled while keeping the same distribution and average. The standard error of the individual objective component also increases in general. However, the objective function, a weighted average of performance measures, can be better or worse than before due to randomness of variables and probabilistic design in the algorithm.

The patient flow sequences of the ophthalmology clinic depicted in Table 9 could exhibit some deviation even for patients in the same class. This depends on an individual's health condition and the on-site assessment of the health professional. To reduce the problem complexity, the given information (Table 9) has been used only in this study. (For information updates, the patient flow sequence and waiting times could be recorded with patients' consent and information technology support.) The current results are based on a computational approach with no guarantee of optimality or proof of stochastic convergence. The weights of the multiple objectives, representing their relative importance, have been chosen on the basis of easily perceived (Section 4.3) but not exhaustive scales. (Some guidelines on the choice of objective weights are given in Section 5.2.) The resource allocation phase (stage I) relies on the existence of multiple skills for certain staff (Table 6) to allow reassignment between procedures. Thus for other specialty clinics or an environment with multiple specialties sharing some common resources or waiting areas, the best combination of integrated strategies would need to be investigated for their specific parameters (Tables 3–6 and 9) and characteristics.

The public hospital in our study is currently employing electronic monitoring and reactive control to shorten waiting time. They have developed an electronic management system that displays the real-time queue status of specialist outpatients, informing them about their expected waiting time. If actual waiting times were long, more staff with lighter workload at the time would be called to the clinic. The operations manager of the specialist outpatient departments has regarded our research results and conclusions as specific and clear. Despite not having specific plan of implementation yet, we anticipate there would be comprehensive planning with similar technology tools in the future.

## 6. Conclusion

This article elaborates one of the first studies analysing an integrated resource allocation and (block) appointment scheduling problem for tactical and operational planning. Developing novel multidecision solution approaches to better address real-life problems is a research direction suggested by a recent survey of outpatient appointment systems [9]. From the observation that patient demand (*Q*) is growing while staff supply is often a shortage issue in healthcare organizations, our study proposes long-term and short-term strategies summarized as follows:

1. Long-term strategy: The bottleneck procedure may not be the most obvious one expected (e.g., doctor's consultation). This study has illustrated a computational approach to identify multiple bottleneck procedures in an ophthalmology clinic. Resource flexibility at the bottleneck procedures in the clinic can be increased by providing staff training to enable redeployment or by employing automation.
2. Short-term strategy: Integrating strategies can effectively improve system performance. In this study, a two-stage model integrating the greedy resource allocation strategy and the adaptive appointment scheduling heuristic [2] extended by simulation shows the most promising improvement on the patient- and staff-centered performance measures. Simply combining the resource allocation strategy with a good priority rule ((2) and (3), CP, LNS, or SPT) can bring about a significant improvement over the base scenario (with a given resource configuration, appointment schedule, and the FCFS rule).

Future studies could examine other specialty clinics or an environment with multiple specialties sharing some common resources or waiting areas. Another direction is to customize the integrated strategies for implementing continuity of care. Trade-offs in the objectives can then be evaluated with the current setting where resources are pooled.

## Figures and Tables

**Table 1:** Methodologies for analysis.

Type	Resource allocation (stage I)/appointment scheduling (stage II)	Service discipline
Base scenario	(Given plan and schedule)	FCFS
Integrated strategy	Stage I + II (Sections 3.1 and 3.2)	Adaptive rule, priority rules (Sections 3.2.2 and 3.4)
	Stage I (Section 3.1)	Adaptive rule, priority rules (Sections 3.2.2 and 3.4)

**Table 2:** Operational information and parameters in an outpatient (block) appointment system.

Patients
Number of appointments per session
Number of patient classes
Set of patient class and distribution
Number of paths by patient class
Set of paths by patient class
Distribution of visitors per patient
Patient punctuality
Probability of arriving early/late
Distribution of earliness by patient class
Distribution of tardiness by patient class
Resources
Resource groups (resource units in group)
Available start time by resource unit
(Initial) allocation plan of resource units to procedures
Skill set by resource unit
Procedures
Number of procedures
Set of procedures, operating mode and capacity (single/batch)
Procedure duration by patient class
Movement time between successive procedures (including record handling)
Operating environment
Duration of appointment session
Number of time blocks in appointment session
Start time of time blocks
Appointment schedule
(Initial) distribution of appointments by time block
Minimum number of appointments per time block

**Table 3:** Parameter values used in experiments from an ophthalmology clinic [1, 2].

Patients
250 appointments per session
4 patient classes and 8 paths (with details in Table 9)
Distribution of visitors per patient = {0 (70%), 1 (23.3%), 2 (6.7%)}
Patient punctuality
Probability of arrival status = {0.7 (early), 0.3 (late)}
Empirical data on earliness (min)
Patient class 1 to 3: {1, 8, 10, 12, 13, 14, 15, 16, 24, 30, 37, 47, 80, 121};
Patient class 4: {13, 20, 21, 25, 27, 31, 32}
Empirical data on tardiness (min)
Patient class 1 to 3: {0, 6, 8, 8, 11, 16}
Patient class 4: {3, 6, 30, 59}
Resources
Doctors (D1,…, D8), nurses (N1,…, N16), educational video (TV)
Available start time (in minute) = {30 for doctors, 0 otherwise}
(Initial) allocation plan of resource units to procedures (Table 4)
Skill set by resource unit (assumption in Table 6)
Procedures
A total of 8 procedures
Set of procedures, operating mode and capacity (single/batch) (Table 5)
Procedure duration by patient class (Table 5)
Movement time between successive procedures (Table 5)
Operating environment
4.5 hours (or 270 min) of appointment session
12 time blocks in appointment session
Start time of time blocks (in minute) = {0, 15, 30, 45, 60, 75, 90, 105, 120, 135, 150, 165}
Appointment schedule
(Initial) distribution of appointments = {16, 41, 19, 38, 19, 20, 23, 19, 14, 15, 13, 13}
A minimum of 6 appointments per time block

**Table 4:** (Initial) allocation plan of resource units to procedures.

Resource group	I Consultation	II Nurse assessment/health consultation	III Visual acuity/eye exam	IV Measure eye pressure/apply eye drops	V + VI Registration + appointment booking	VII Nurse reminder on day surgery	VIII Educational session
Doctors	D1–D8						
Nurses		N1, N2	N3–N9	N10–N12	N13, N14 (patient classes 1 and 4) N15 (patient classes 2 and 3)	N16	
Educational video							TV

**Table 5:** Procedure duration (min) by patient class, operating mode, and capacity.

Patient class	I Consultation	II Nurse assessment/health consultation	III Visual acuity/eye exam	IV Measure eye pressure/apply eye drops	V Registration	VI Appointment booking	VII Nurse reminder on day surgery	VIII Educational session	Movement time between procedures (incl. records)
1	5–10	4–6	3–5	3–5	1–1.5	1–1.5	—	—	2-3
2	5–10	4–6	3–5	2–4	3–5	1–1.5	—	—	2-3
3	5–10	4–6	3–5	2–4	2–4	1–1.5	—	—	2-3
4	5–10	4–6	3–5	3–5	1–1.5	2-3	5–10	8–12	2-3
Operating mode (capacity)	Single (1)	Single (1)	Single (1)	Single (1)	Single (1)	Single (1)	Single (1)	Batch (15)	—

**Table 6:** Resource skill set (resource flexibility assumption).

	Doctors	Nurses						Educational video
Resource unit	D1–D8	N1, N2	N3	N4–N9	N10–N12	N13–N15	N16	TV
Procedure	I	II	II, III, IV	III, IV	IV	V, VI	VII	VIII

**Table 7:** Algorithm parameters.

Description	Notation	Value
Number of simulation replications per schedule	*n*	30
Maximum % estimation error in *Z* (for determining *n*)	*ε*	10%
Level of confidence in estimating *Z* (for determining *n*)	1−*α*	95%
Level of significance in testing for an improved schedule	*γ*	0.1
Number of new schedules created from an incumbent schedule	iter_max_	5
Initial pool size of patients for rescheduling	*p*_0_	10
Fixed increment of pool size	*p*_step_size_	2
Initial maximum pool size	*p*_max_size_	12
Maximum time limit for algorithm (CPU seconds)	*t*_lim_	7200

**Table 8:** Multiobjective performances of different integrated strategies under the resource flexibility scenario where (*w*_1_, *w*_2_, *w*_3_) = (1, 10, 1/2).

Integrated strategy	Patient selection rule	Solution	*Z*	*Z*_1_: avg. patient waiting time (min)	*Z*_2_: avg. resource overtime (min)	*Z*_3_: avg. congestion per time unit	Avg. max. resource overtime (min)
(Base scenario) None	FCFS	—	1408	152	122	78	193
Stage I only	FCFS	Best	597	138	44	34	132
Stage I + II	FCFS	Best	508	145	34	37	140
Stage I + II	Eqns. (2) and (3)	First	449	194	20	118	178
		Best	254	140	9	42	109
Stage I + II	CP	First	606	198	34	127	179
		Best	263	146	9	47	109

**Table 9:** Patient classes and paths.

Class (%)	Description	Path number (%)	Path
1 (54%)	Old (or continuing) cases	1 (66.67%)	Registration → visual acuity → measure eye pressure/apply eye drops → doctor's consultation^∗^ → appointment booking
		2 (33.37%)	Registration → visual acuity → measure eye pressure/apply eye drops → doctor's consultation^∗^ → health consultation

2 (27%)	New cases	1 (92.5%)	Registration → nurse assessment^∗^ → appointment booking
		2 (7.5%)	Registration → nurse assessment^∗^ → visual acuity → measure eye pressure → nurse assessment → appointment booking

3 (10%)	Enquiry cases	1 (10%)	Registration → nurse assessment^∗^ → leave
		2 (72%)	Registration → nurse assessment^∗^ → visual acuity → measure eye pressure → nurse assessment → appointment booking^#^ → apply eye drops → doctor's consultation
		3 (18%)	Registration → nurse assessment^∗^ → visual acuity → measure eye pressure → nurse assessment → appointment booking^#^ → doctor's consultation

4 (9%)	Day surgery cases	1 (100%)	Registration → eye examination → apply eye drops → educational TV session → doctor's consultation → appointment booking (surgery operation) → nurse reminder

^∗^First diversion. ^#^Second diversion.
